# Silicon Oxycarbide and Silicon Oxycarbonitride Materials under Concentrated Solar Radiation

**DOI:** 10.3390/ma14041013

**Published:** 2021-02-21

**Authors:** M. Alejandra Mazo, Isabel Padilla, Aurora López-Delgado, Aitana Tamayo, Juan Rubio

**Affiliations:** 1 Ceramics and Glass Institute, CSIC, Kelsen 5, 28049 Madrid, Spain; aitanath@icv.csic.es (A.T.); jrubio@icv.csic.es (J.R.); 2 National Centre for Metallurgical Research, CSIC, Av. Gregorio del Amo 8, 28040 Madrid, Spain; isapadilla@cenim.csic.es (I.P.); alopezdelgado@cenim.csic.es (A.L.-D.)

**Keywords:** silicon oxycarbide, silicon oxycarbonitride, concentrated solar radiation, high temperature solar receivers, thermal shock test

## Abstract

The potential application of silicon oxycarbonitride (SiOCN), silicon oxycarbide (SiOC) and silicon oxycarbide–SiC (SiOC–SiC) for photothermal devices such as volumetric solar absorbers has been studied evaluating the response to thermal shock from a Fresnel lens. The accelerated ageing test comprises fast heating (32 °C min^−1^) and cooling rates (27 °C min^−1^) from 100 to 1000 °C and dwelling times of 10 min. Porous materials (SiOCN_p_ and SiOC_p_) failed the thermal shock tests; they were massively degraded by the formation of a large depression in the focus of solar radiation. Dense materials (SiOC_d_ and SiOC–SiC_d_) withstood 100 cycles of thermal shock ageing tests due to the formation of a protective silica layer. The absorptance values for dense materials remained fairly constant before and after thermal shock tests: from 94.5 to 94.3% for SiOC_d_ and from 93.3 to 93.3% for SiOC–SiC_d_. These preliminary studies indicate their potential for high-temperature solar receiver applications.

## 1. Introduction

The huge demand for energy and the global warming of the planet have made the development of competitive energy generation from non-polluting renewable sources (solar, wind, biomass, etc.) an urgent necessity. Concentrated Solar Power (CSP) systems are currently an important focus of interest for their advantages over other power generation sources. They allow energy to be affordably stored using energy storage systems so electricity is available when there is no sunlight (at night or on cloudy days) [1] and can be used in conjunction with other energy systems [2].

However, their great handicap is their high cost, which can be solved by enhancing the efficiency of the process by increasing the working temperature to as high as possible (>700–1000 °C) [3,4,5]. Solar power tower is the most promising CSP system as it enables the highest working temperatures (>1000 °C, and basically limited by the degradation of the materials) and thus improves the efficiency of the process [3,5]. In terms of the operational conditions in CSP tower systems, the solar receiver is the main component in the material degradation, as it is exposed to highly concentrated solar radiation and high temperatures, which are the main ageing factors. The receivers are subjected to thermal shock from major temperature variations between day and night and by the appearance of clouds [6].

Materials used as high temperature solar receivers must satisfy several properties (physical, chemical and mechanical) which can vary depending on the specific type of receiver and solar tower configuration. For good heat transfer to the fluid, and in order to minimize contraction/expansion due to temperature fluctuations, solar receivers should have good thermal conductivity (K) and a low coefficient of thermal expansion (CTE). They must also have creep resistance at high temperature, toughness and high resistance to oxidation and corrosion due to very harsh operational conditions [4]. Of course, low cost and long service life are also required. There is an urgent need to research and develop materials that fulfil all these properties and increase operational temperature and efficiency in order to ensure the economic sustainability of concentrated solar tower technology. Two kinds of materials are generally widely used for these purposes: metals and ceramics [4,7]. As metals, Ni alloys (i.e., INCONEL) are common due to their exceptional mechanical properties up to 700 °C. Metal receivers require a coating, usually Pyromark^®^ black paint, in order to increase solar absorptance, which also limits its utilization up to 650 °C due to the thermal degradation [8]. Ceramics such as SiC can withstand operational conditions of >1000 °C, and have good thermal conductivity and resistance to oxidation and corrosion and high mechanical strength to high temperature, among other properties [9], although they are unfortunately limited by their fragility which can cause the system to fail during service [10].

A large number of studies are currently focused on the reliability and long-term stability of CSP components, especially receivers, during service in order to determine the commercial and industrial suitability and viability of both the receiver and new candidate materials. These studies are based on numerical models and predictions which take into account material properties and other characteristics of receiver design as well as ageing factors and should be completed with accelerated ageing tests that can evaluate the receiver behaviour under real operational conditions [6,7,10,11,12,13,14,15,16]. The important and not easy issue is to develop testing methods and standards of validation of materials employed for solar energy applications, which is usually limited by solar testing facilities and also characterization methods available within laboratories [16,17]. The first attempts were made by Rojas-Morín and Fernández-Reche [11] employing a parabolic dish over INCONEL determining both a numerical model and the fatigue mechanical behaviour. More recently, a solar furnace was employed for testing potential candidates after the exposition to thermal cycles by direct concentrated solar irradiation. Vidal and Martínez [16] studied the weight loss, visual appearance, reflectance and phase composition by X-ray diffraction (XRD) of aluminium nitride, alumina, zirconia and alumina/zirconia. Oliveira et al. [14] compared the evolution of mechanical properties and the suitability of experimental data with Gibson-Ashby model of mullite, brown alumina and ceria based materials. Morris et al. [4] studied the behaviour of metallic and intermetallic alloys using a Fresnel Lens. Recently, Mazo et al. [18] analysed the thermal shock behaviour of ceramic materials (i.e., SiC and silicon oxycarbide (SiOC)) using the same solar concentrator facility. Palacios et al. [15] carried out an isothermal ageing treatment over the samples (SiC, sand and Fe_2_O_3_) and studied the changes by the variation of colour, specific heat capacity, absorptance and XRD. Finally, a solar accelerated ageing facility was employed by Lalau et al. [7], who studied the ageing of INCONEL and SiC exposed to thermo-mechanical stresses employing a numerical model and a non-destructive acoustic-emission technique.

Based on previous results [17,18], this work proposes the use of SiOC and silicon oxycarbonitride (SiOCN) materials for their potential as high temperature solar receivers. SiOC materials have interesting intrinsic properties such as good resistance to oxidation in harsh environments, high and tunable mechanical properties, low creep, high absorbance, low CTE, in addition to tunable thermal and electrical properties [18,19,20,21,22,23,24]. It is important to note that many of these properties can be designed and adjusted simply by varying the composition, microstructure and processing conditions such as temperature and type of sintering. SiOC materials are formed by a Si–O–C mixed network and graphite-like carbon phase (known as C_free_) embedded homogeneously inside them. At temperatures over 1200 °C, the material evolves SiO_2_, SiC and highly disordered graphite-like carbon. As the temperature rises, SiC and C_free_ reorganize into more ordered species, although SiO_2_ remains amorphous [25].

In this work preliminary experiments on the behaviour of novel promising candidates for solar receivers have been carried out., but another experiments are required in other to ensure the suitability at industrial scale. In the earlier studies [18], porous SiOC (SiOC_p_) underwent a massive degradation of its surface at 1200 °C during the first cycles of accelerated solar ageing, making it necessary to test its suitability at lower temperatures (i.e., 1000 °C). Due to their very high resistance at 1200 °C, further insight is required of the behaviour of dense SiOC materials (SiOC_d_) at lower temperatures (i.e., 1000 °C) in order to complete the study of their thermal shock resistance. Moreover, it is well known that Si_3_N_4_, Si_2_N_2_O and their derived materials have attractive properties (high temperature and oxidation resistance, good mechanical properties, low creep, resistance to thermal shock, etc.) [26,27], that make them suitable for high-temperature structural applications. The new study is therefore proposed of porous silicon oxycarbonitride (SiOCN_p_) materials with Si_2_N_2_O and/or Si_3_N_4_ phases as candidate materials for use as high-temperature solar receivers, with an assessment of their response when subjected to thermal shock tests at 1000 °C. Finally, dense SiOC–SiC composites (SiOC–SiC_d_) are also evaluated due to the well-known and exceptional properties of SiC and its widespread use as a high-temperature solar receiver [10] in combination with the intrinsic properties of SiOC.

## 2. Materials and Methods

### 2.1. Materials

Four samples were investigated under an accelerated ageing test: two porous materials, SiOCN_p_ and SiOC_p_ and two dense materials, SiOC_d_ and SiOC–SiC_d_. The raw material in all of them was composed by tetraethylortosilicate (TEOS) and polydimethysiloxane (PDMS) silanol terminated organic-inorganic hybrids synthesized by the sol-gel method [28]. The porous SiOCN_p_ material was obtained from this TEOS/PDMS hybrid in a two-step process of nitridation at 750 °C (3 h) under N_2_/NH_3_ (3:1) atmosphere, and subsequent pyrolysis under N_2_ at 1100 °C (2 h). The chemical composition of this SiOCN_p_ material is SiO_1,47_C_0,11_N_0,23_ and the porosity 80%. The porous SiOC_p_ material was obtained by one-step pyrolysis under N_2_ atmosphere at 1100 °C (2 h) of the TEOS/PDMS hybrid [29]. The chemical composition of this SiOC_p_ is SiO_1.67_C_0.50_ and the porosity 81%. Dense SiOC_d_ and SiOC–SiC_d_ samples were obtained by sintering fine SiOC powder mixtures by spark plasma sintering at 1500 °C, 40 MPa (SPS, Dr. Sinter, SPS-510CE, SPS Syntex Inc., Kanagawa, Japan). Two types of SiOC powders were used: pure SiOC powders (surface area = 6 m^2^ g^−1^, mean particle size d_50_ = 3.4 μm and composition SiO_1.69_C_0.43_), and a mixture containing a 10 wt(%) of SiC powders (SiOC–SiC) generously supplied by Advanced Thermal Devices, S.L., Madrid, Spain; surface area = 33 m^2^ g^−1^; mean particle size d_50_ = 0.9 μm and composition: SiC = 97.8%; SiO_2_ = 1.6%; C = 0.3%). A detailed description of the experimental procedure can be found elsewhere [28,30]. The initial porosities of SiOC_d_ and SiOC–SiC_d_ materials were 3 and 4%, respectively and K were 1.432 and 2.143 W m^−1^ K^−1^, respectively. SiOCN_p_ had a quadrangular prism shape with approximate dimensions of 20 mm × 20 mm × 10 mm, while the other samples were cylindrical; SiOC_p_ was 30 mm × 10 mm and both SiOC_d_ and SiOC–SiC_d_ were 20 mm × 3 mm.

### 2.2. Solar Thermal Shock Tests

The accelerated ageing test involved a thermal shock test under concentrated solar radiation using a Fresnel lens. A detailed description of the concentrated solar facility was previously reported [4,18]. To reach the desired temperature and maintain it throughout the test time, the experiments were carried out on sunny and unclouded days between 10:00 and 14:00 (apparent solar time), when levels of direct solar irradiance were close to 1000 W m^−2^. The Fresnel lens has a power density of 260 W cm^−2^, which allows a concentration of solar radiation to be reached of more than 2600 times the incident solar radiation [31]. The samples were placed in a stainless steel sample chamber and a type R thermocouple was placed over the sample precisely at the focus point of maximum solar radiation in order to track the changes in temperature.

Each thermal shock cycle consisted of fast heating/cooling stages by covering or uncovering the Fresnel lens. The cooling was passive, the sample was in contact with the air at the atmospheric conditions and no other cooling procedures were used. Each cycle involved a heating of the sample from initial temperature to 1000 °C, maintaining it for 10 min, and then allowing it to cool to 100 °C. The average heating rate was 32 °C s^−1^ and the average cooling rate was 27 °C s^−1^. Initially 25 thermal shock cycles were planned for each sample, then depending on whether the sample withstands these cycles (i.e., no breakage or massive degradation is detected by visual inspection), the cycles were continued up to a maximum of 100.

### 2.3. Characterization Techniques

The variation in the material surface before and after the thermal shock test was observed with several techniques. Topography and roughness were measured using an interferometric confocal microscope (ICM) (PLμ 2300 Optical Profile, Sensofar, Barcelona, Spain), using 20 × EPI and 50 × EPI optical objectives with a field of view of 625 µm × 468 µm and 273 µm × 205 µm respectively. The roughness value (*R_a_*: arithmetic average value of roughness) (ISO 4287/1 and DIN 4768) was determined by analysing three different zones placed at three different distances from the focus centre: the nearest focus area (at 2–3 mm from the focus centre), an intermediate zone (middle zone, at 5–6 mm from the focus centre) and, the furthest area (at 12–13 mm from the focus centre). As we stated in a previous work [18], there is a thermal gradient of at least 500–600 °C from focus to the most distant areas of the tested sample, so small variations (few millimeters) from focus zone lead to great differences in temperature.

*R_a_* values were referred as *R*_0_ = initial value before and after the test, *R*_1_ = focus zone, *R*_2_ = intermediate zone and *R*_3_ = far from the focus zone. Pristine and tested surfaces were also analysed by scanning electron microscopy (SEM, TM1000, Hitachi High-Technologies Corp., Tokyo, Japan). Infrared spectra in the attenuated total reflection mode (ATR) were performed on a spectrometer using a MIRacle™ device (IR Spectrum BX, Perkin Elmer Inc., Waltham, MA, USA). Raman spectra were recorded on a spectrometer (In Via, Renishaw, Gloucestershire, UK) with an excitation wavelength of 514 nm. The in-plane lateral domain size of the C_free_ phase (L_a_) was calculated according to (1) [32]:

I_D_/I_G_ = C(λ)/L_a_ (nm) (1)

where I_D_/I_G_ is the intensity ratio of D and G bands and C(λ) is a constant which acquires a value of 4.4 when a laser of 514 nm is used in Raman measurements. The spectral reflectance (R) from 400 to 1100 nm at R.T. is measured based on the Standard ASTM E 424−71 [33] using a spectrometer (UV/Vis Lambda 40, Perkin Elmer Inc., Waltham, MA, USA) with integrating sphere. The solar reflectance was computed by integrating the solar spectrum based on the Standard ASTM G 173. The solar absorptance (*A*) of the materials was calculated as *A = 100* *−* *R* in %, as it is more obvious for opaque materials. Real density values were determined with a pynometer (AccuPy 1330, Micromeritics Instrument Corp., Norcross, GA, USA) using He, and apparent density values were determined by measuring the weight and dimensions of the samples (geometrical density). Porosity (*P*) was calculated with both apparent and real density values following (2).

P(%) = (1 − Apparent density/Real density) × 100(2)

K was determined at R.T. employing a TCi thermal conductivity analyzer (Mathis Instruments, C-Therm Technologies, Fredericton, Canada).

## 3. Results and Discussion

Table 1 shows the roughness values of pristine samples, the surface topography is studied by SEM (Figure S1) and ICM (Figure S2) images. Based on all of them, it is concluded that SiOCN_p_ is the most porous material formed by spherical-shaped interconnected particles with pores in the macropore range around 20 μm. The confocal image and R_a_ follow the same trend with an average of 20 μm. SiOC_p_ displays the same porous morphology but the pores are smaller (10–15 μm) [29], and R_a_ is therefore somewhat smaller, with a value of 5.4 μm. The dense samples (SiOC_d_ and SiOC–SiC_d_) have homogeneous and fine topographies with some pull-outs and polishing scratches, especially in the case of the SiOC–SiC_d_ sample. Confocal images and R_a_ values show the same results, with very low roughness values (SiOC_d_ = 0.2 μm and SiOC–SiC_d_ = 0.4 μm).

### 3.1. SiOCN_p_

**Figure 1:**
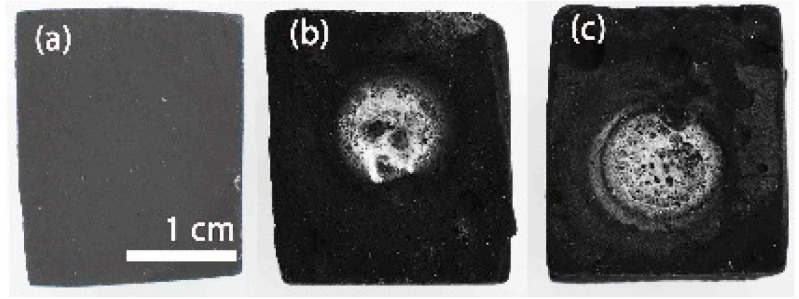
SiOCN_p_ sample (**a**) before; (**b**) after the first and (**c**) second cycle of the thermal shock test.

Figure 1a shows the initial appearance of the SiOCN_p_ sample before the thermal shock experiment. As can be clearly seen in the temperature-time graph for this sample (Figure S3a), in the first cycle, the temperature increased uncontrolled to 1200 °C after a very few seconds and the application of solar radiation was interrupted by covering the Fresnel lens, as can be observed by the sharp drop in temperature. The direct observation of the sample showed the formation of a large bubble, located precisely in the focus zone, which, when broken, reveals a whitish melted layer covering the resulting valley (Figure 1b). To confirm this result, a second thermal shock cycle was performed on the other side of the SiOCN_p_ sample and the same behaviour was found (Figure 1c). In this second experiment the temperature increased up to 1000 °C (Figure S3a) and a new bubble was formed, which exploded after 90 s, generating a large crater covered with a whitish melted layer. A weight decrease of 18% was also observed after these thermal shock cycles, and the porosity of the sample varied from 80 to 79%.

The weight loss must be ascribed to the oxidation of the C_free_ phase (3), although in order to explain all the changes observed the oxidation of other species such as Si_3_N_4_ (4), Si_2_N_2_O (5) and SiOC (6), which are also present in SiOCN materials cannot be ruled out [34,35]. Reaction (3) usually occurs from 400–800 °C and the other reactions at higher temperatures from 800–1000 °C. [34,35] CO_x_ refers to CO and CO_2_.

C_free_(s)+O_2_(g) → CO_x_ (g) (3)

Si_3_N_4_(s)+3/4O_2_(g) → 3/2Si_2_N_2_O(s)+1/2N_2_ (g) (4)

Si_2_N_2_O(s)+3/2O_2_(g) → 2SiO_2_(s)+N_2_ (g) (5)

SiOC(s)+O_2_ (g) → SiO_2_(s)+CO_x_ (g) (6)

The oxidation of SiOCN materials obeys a very complex mechanism as it involves the formation of protective layers of SiO_2_ and Si_2_N_2_O ((4), (5) and (6)) that limit the inward diffusion of O_2_ and the outward diffusion of N_2_ and CO_x_, and the decomposition of C_free_ (3), which produces additional CO_2_ [35]. In any case, due to the highly porous microstructure of the SiOCN_p_ material and the very severe thermal shock conditions, the inward diffusion of O_2_ and the outward diffusion of N_2_ and CO_x_ occur practically without restriction before the protective layer is well formed, so the material is massively degraded during the first thermal shock cycle.

**Figure 2:**
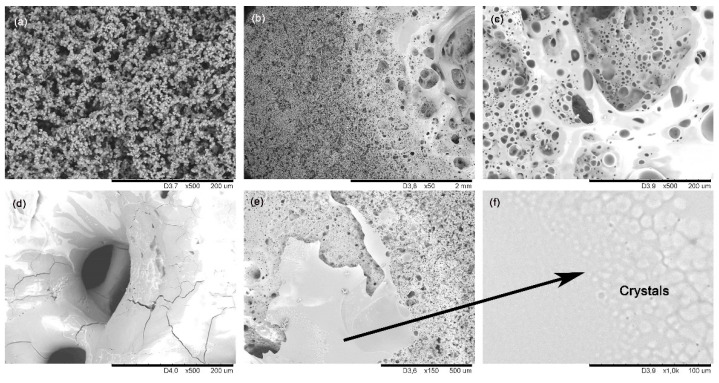
SEM images of the initial SiOCN_p_ surface and after the first thermal shock cycle under concentrated solar radiation by Fresnel lens: far from the focus zone (**a**); focus zone (**b**) large hole (**c**) large bubbles, (**d**) melted zone (**e**) dense layer, (**f**) crystallizations.

SEM images indicate the SiOCN_p_ material remains unaltered far from the focus zone (Figure 2a), and has the same appearance as the initial SiOCN_p_ (Figure S1a); however, in the focus zone precisely, the material is deeply degraded and a large hole appears (Figure 2b). Large bubbles can be observed in this zone (Figure 2c), probably due to develop of gaseous species formed during testing, and a melted zone is also observed (Figure 2d); both these effects are partially interrupted by the presence of larger pores and cracks (reactions 3–6). The formation of a dense layer is also observed in another zone (Figure 2e), along with the presence of crystallizations (Figure 2f). In this case, it does not act as a protective layer; although it is dense, is not tightly bonded to the material and does not cover the whole surface homogeneously. The gaseous species (i.e., O_2_ inward and N_2_ and CO_x_ outward) can freely pass through it without restriction, which can probably be attributed to both the high porosity of the SiOCN_p_ material and to the very fast and severe conditions of the thermal shock test.

**Figure 3:**
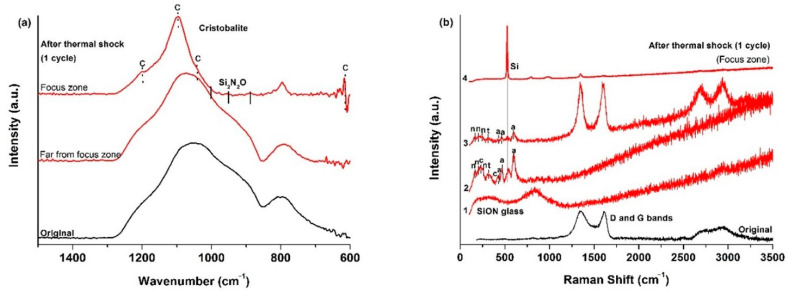
(**a**) ATR; (**b**) Raman spectra of the initial SiOCN_p_ surface, and after the first thermal shock cycle under concentrated solar radiation by Fresnel lens. Si = metallic silicon, n = Si_2_N_2_O, t = tridymite, c = cristobalite, a = amorphous silica. 1–4 refer to different zones in the focus zone after thermal shock.

The ATR spectrum (Figure 3a) of the initial SiOCN_p_ material displays a very broad signal from 850 to 1275 cm^−1^, which includes the absorption bands of both Si–N (850 cm^−1^ [36]) and Si–O bonds (1072 cm^−1^ [19]) and other smaller bands located at 800 cm^−1^ associated to the symmetric stretching of Si–O bonds and to the SiOC mixed network [37]. The broadening of this first signal must be associated to the Si_2_N_2_O species generated with a different N/O ratio during the nitridation process [36]. After the first thermal shock test cycle there are zones where no change is detected (far from focus zone), although there is one zone, in the focus zone precisely, where bands related to cristobalite appear (intense sharp bands at 1202, 1095, 1035 and 619 cm^−1^ [38] and to Si_2_N_2_O_2_ with a different N/O ratio (900, 950 and 1000 cm^−1^ [36]).

The Raman spectrum of the initial SiOCN_p_ (Figure 3b) material shows the D and G bands related to highly disordered carbon-derived materials associated to the C_free_ phase [39]. The L_a_ value is 4.0 nm. After the thermal shock tests, the sample surface in the focus zone changes radically, revealing the complex nature of the SiOCN transformation (Figure 3b). Spectrum 1 shows the presence of a SiON glass (broad bands centred at ≈300 and ≈850 cm^−1^) [40]. Spectrum 2 indicates the presence of a mixture of silica-derived compounds such as cristobalite (230 and 415 cm^−1^), amorphous silica (429, 487 and 606 cm^−1^), tridymite (345 cm^−1^) [38,41] and crystalline Si_2_N_2_O (172–184, 230–250 cm^−1^) [40]. The presence of D and G bands related to C_free_ in Spectrum 3 is also noticeable, and probably indicates that some carbon particles may become trapped inside the melted layer due to the very rapid degradation of the material and the formation of a SiO_2_/Si_2_N_2_O layer. Finally, a very sharp and intense peak centred at ≈520 cm^−1^ can also be seen in Spectrum 4, which could be associated to Si produced as a result of the decomposition of the SiOCN_p_ material and the very severe conditions of the thermal ageing test [40]. All these findings are in agreement with the melted zone and crystallizations observed by SEM (Figure 2).

These results confirm the formation of a layer of SiO_2_ (mainly amorphous silica and cristobalite) and crystalline Si_2_N_2_O, and probably the formation of metallic Si due to the decomposition of SiOCN during the severe conditions of the thermal shock tests. In any case, the SiOCN_p_ material does not pass the thermal shock test, but undergoes rapid and severe oxidation and decomposition. These materials are not suitable for high-temperature solar receiver applications. However, the very rapid formation of the SiO_2_/Si_2_N_2_O “protective” layer highlights the potential of this material. To be used for this purpose, the material’s porosity would have to be eliminated in order to minimize the inward/outward fluxes of gases that produce the rapid and massive degradation, and the formation of a dense and tightly-bonded protective layer.

### 3.2. SiOC_p_

**Figure 4:**
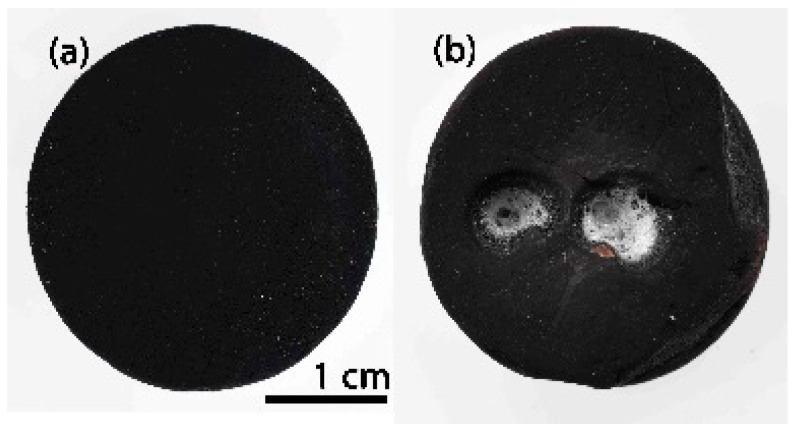
SiOC_p_ sample (**a**) before; (**b**) after 38 cycles of thermal shock tests.

The initial appearance of the SiOC_p_ material is shown in Figure 4a. Figure S3b shows the temperature recorded throughout the experiment (38 cycles) is constant, although there are some temperature variations caused by fluctuations in the direct solar irradiance and the wind, due to the fact that the thermal shock experiments are performed outdoors. After 25 thermal shock cycles the appearance of the SiOC_p_ material remains unchanged and no sign of degradation is detected. The study by ICM also confirms that the topography of the SiOC_p_ remains unaltered, showing the same surface regardless of the area studied (Figure S4). Only a slight increase in the roughness values is observed compared to the initial values of the SiOC_p_ sample (Table 1). In view of the results obtained, another set of thermal shock cycles was conducted, and after cycle 37 a large crater appeared covered with a white melted zone on the black surface precisely in the focus zone. Another cycle was done for confirmation (cycle 38th), and the same result was achieved (i.e., the formation of a new crater) (Figure 4b. A weight decrease of 5% was observed after the thermal shock cycles, and the porosity of the sample varied from 81 to 82%. The weight decrease is due to C_free_ degradation (3), although the formation of a white melted zone can be associated to the SiOC decomposition (6) and the subsequent formation of a SiO_2_ layer.

**Figure 5:**
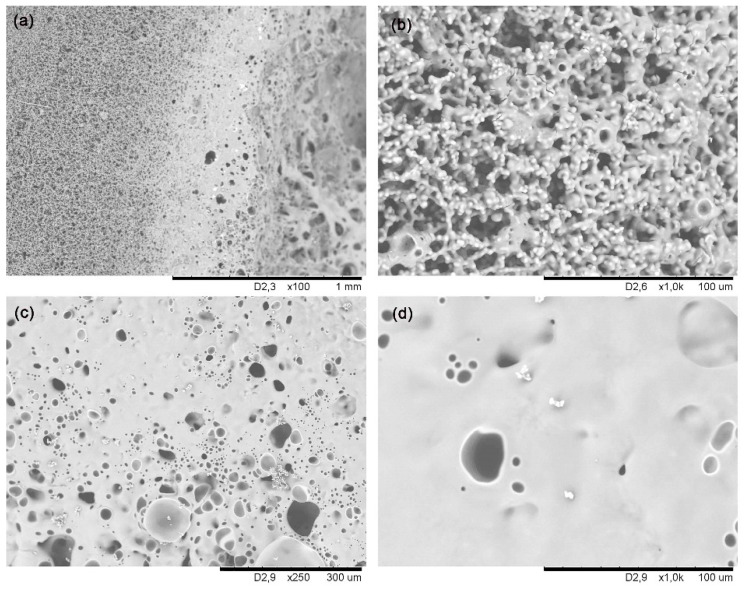
SEM micrographs of the SiOC_p_ surface after 38 cycles of thermal shock tests using concentrated solar radiation with the Fresnel lens: (**a**) global view; (**b**) melted and pristine material of medium distance area; (**c**,**d**) melted area nearest the focus of the solar radiation.

SEM images after 38 cycles are shown in Figure 5. The material surface underwent severe degradation as a result of the thermal shock tests. The zone farthest away from the focus remains unchanged (Figure 5a). But as the distance from the focus decreases, there first appears a mixture of pristine material and new melted material (Figure 5b); and then a dense layer can clearly be seen in the focus zone itself (large crater covered with a layer of white melted material; see Figure 4b), partially interrupted by bubbles due to the gases evolved during the C_free_ (3) and SiOC (6) degradations (Figure 5c,d). As the material decomposes (i.e., C_free_ and SiOC) due to the very harsh experimental conditions and to the highly porous microstructure of the material, O_2_ can easily diffuse inside the material and degrade both C_free_ and SiOC phases, producing gaseous (CO_2_ and CO) and solid species (melted SiO_2_) at the same time. Most of these gaseous species can also diffuse outwards, but some become trapped inside the recently formed molten layer (Figure 5c,d).

**Figure 6:**
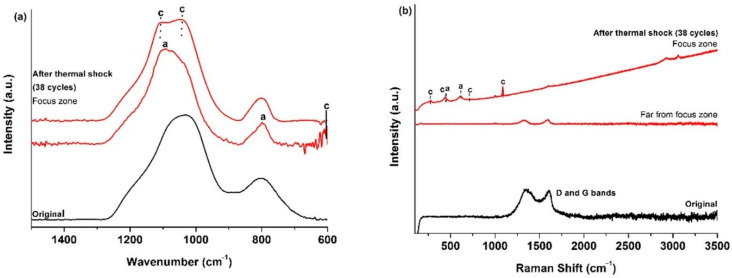
(**a**) ATR; (**b**) Raman spectra of the initial SiOC_p_ surface, and after 38 thermal shock cycles under concentrated solar radiation by Fresnel lens near and far from the focus zone. c = cristobalite, a = amorphous silica.

The ATR spectrum of the initial SiOC_p_ material (Figure 6a) shows the typical features of these materials (1050 cm^−1^, asymmetric stretching of Si–O [19], 805 cm^−1^ symmetric stretching of Si–O [19] and the SiOC network [37]). In the focus zone, after 38 cycles of thermal shock tests, the stretching of Si–O bonds, especially asymmetric stretching, shifts to a higher wavenumber denoting the formation of both amorphous silica [42] and cristobalite [38] due to the decomposition of the SiOC_p_ material (6). The Raman results perfectly match the results of the ATR spectroscopy (Figure 6b). After the thermal shock test, unaltered zones appeared with only the presence of the D and G bands of the C_free_ phase [39], related to zones far from the focus zone. Bands related to cristobalite (281, 425, 710 and 1083 cm^−1^) and amorphous silica (429, 487 and 606 cm^−1^) [38,43] are observed in other zones close to the solar focus zone. The L_a_ value of the C_free_ phase is 3.5 nm. Similar results were obtained in previous studies on the thermal shock test of SiOC_p_ at 1200 °C, although several differences can be observed, such as the major contribution of ordered species (i.e., cristobalite) and the absence of amorphous silica. In this experiments the sample experienced a noticeable surface degradation just after the first cycle, and a hole was observed to form after five cycles [18]. In any case, the SiOC_p_ material undergoes a massive degradation of its surface, indicating that it is unsuitable for high-temperature solar receivers at least at 1000 °C.

In the case of porous materials, SiOCN_p_ displays the most severe degradation when is compared with SiOC_p_ even if was expected to experience better results due to the formation of a protective layer of SiO_2_/SiO_2_N_2_. This degradation occurs faster in SiOCN_p_ by the presence of bigger pores (SiOCN_p_ ≈ 20 µm, SiOC_p_ ≈ 10–15 µm) which allow gaseous species pass both inward and outward basically without restrictions facilitating the degradation of larger L_a_ domains of C_free_ (SiOCN_p_ L_a_ = 4 nm, SiOC_p_ L_a_ = 3.5 nm).

### 3.3. SiOC_d_

**Figure 7:**
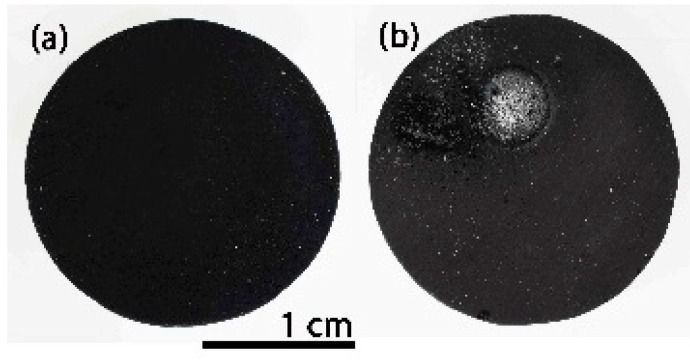
SiOC_d_ sample (**a**) before; (**b**) after 100 cycles of thermal shock tests under concentrated solar radiation by Fresnel lens.

Figure 7a shows the initial appearance of the dense black SiOC_d_ material. As it can be clearly observed in the temperature vs time graph (Figure S3c, during the 100 cycles of thermal shock, there are slight fluctuations in temperature that are related to the experimental conditions; that is, variations in direct solar irradiation due to the presence of clouds and wind, because the tests are performed outdoors [18].

**Figure 8:**
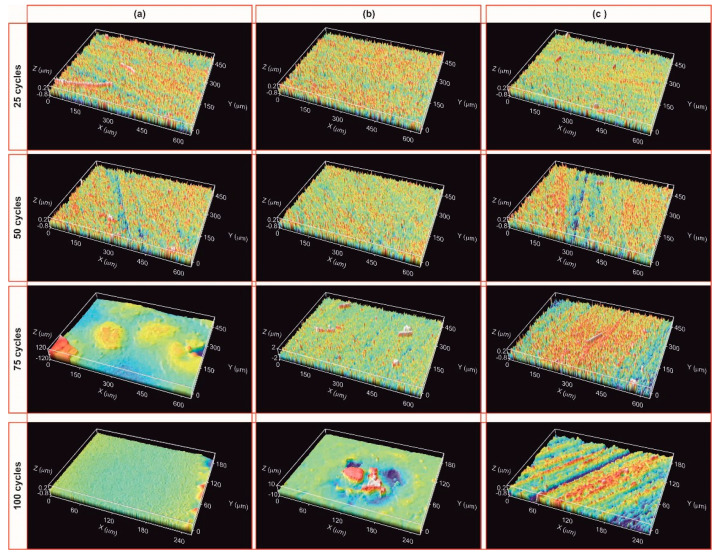
ICM images showing the surface of the SiOC_d_ sample after 25, 50, 75 and 100 cycles of the thermal shock test under concentrated solar radiation by Fresnel lens. (**a**) nearest area, (**b**) medium distance; (**c**) area furthest from the solar radiation focus.

After the first 25 cycles, the visual inspection does not indicate any degradation. The ICM study also confirms this result; the material remains unchanged regardless of the zone analysed (Figure 8a–c (25 cycles)) and the roughness values are also similar to the initial value (Table 1). After 50 cycles, the sample surface displays the same results (Figure 8a–c (50 cycles) and Table 1). After 75 cycles, the sample undergoes a modification in its surface, especially in the zones near the focus of the solar radiation (Figure 8a–c (75 cycles)). High peaks and valleys (>200 μm) appear in the focus zone, while small wires are observed in the intermediate zone (between 22–65 μm in length and 11 μm in diameter). The main change in this area is the appearance of a green-cyan coloured coating probably associated to the formation of a protective SiO_2_ layer [18]. The roughness values agree, and only an increase in the R_a_ value is observed in the focus zone (Table 1). The roughness remains constant in the intermediate zones and away from the focus (Table 1). After 100 cycles, the surface of the sample undergoes additional changes, basically observed in the areas near the focus zone. The focus zone appears smoother, while the intermediate zone appears rougher compared to the image for 75 cycles (Figure 8b). However, the green-cyan coating is still present over the whole surface in this intermediate zone. Again, the zone far from the focus remains almost unchanged. R_a_ values agree with the confocal images, and a significant decrease is only measured in the focus zone, with very low R_a_ values in all the zones. (Table 1). This smoothing was previously attributed to the formation of a melted protective SiO_2_ layer in SiOC materials after the thermal shock ageing test [18]. In previous studies carried out at higher temperature (i.e., 1200 °C), it was observed that the SiO_2_ layer covered the whole surface. In this case, the SiO_2_ only covers the focus zone indicating the huge resistance of SiOC under concentrated solar radiation at 1000 °C after 100 cycles. After 100 cycles of the accelerated ageing test at 1000 °C, the black bulk SiOC_d_ sample displays a whitish halo precisely in the focus zone (Figure 7b) related to the formation of a protective silica layer [18,23]. Weight and porosity do not change, so the material does not experience a massive degradation.

**Figure 9:**
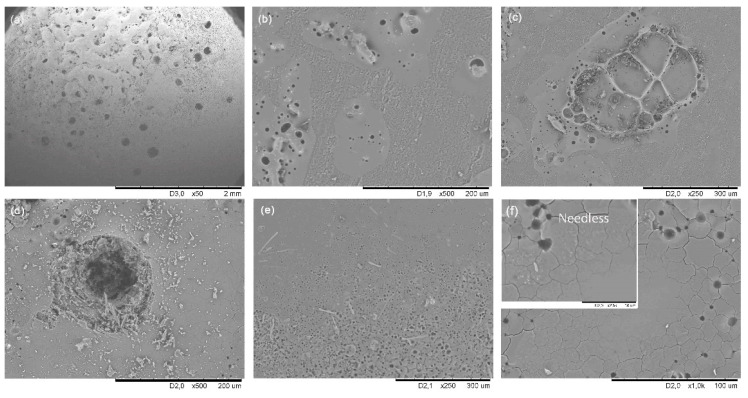
SEM images of the SiOC_d_ sample after 100 thermal shock test cycles: focus zone (**a**) smoothing area; near the focus zone (**d**) valleys (**e**) wires, (**f**) cracks and needless; intermediate zone (**b**) patches without surface modification, (**c**) degraded zones.

SEM images of the SiOC_d_ material surface after 100 cycles of thermal shock at 1000 °C are shown in Figure 9. The focus zone (Figure 9a) reveals a smoothing which agrees with the confocal images and R_a_ values (Figure 8a, 100 cycles and Table 1). As the distance from focus zone increases (downward direction) valleys, wires and cracks appear (Figure 9d–f, respectively) related to C_free_ degradation and the formation of the SiO_2_ layer. The inset in Figure 9f shows the formation of needles inside cracks related to the crystallization of SiO_2_ species (i.e., cristobalite). The intermediate zone (Figure 9b,c) displays patches without surface modification, but isolated degraded zones also appear with the presence of large and small pores surrounded by melted zones. The formation of this inhomogeneous rough surface with melted zones and the remains of exploded bubbles must be associated to the beginning of the surface degradation, (3) and (6) (C_free_ degradation and SiO_2_ formation, respectively). However, it should be noted that both the inward and outward diffusion of gaseous species must be very slow due to the incomplete formation of the silica layer, which is not evident over the whole SiOC_d_ surface as in the case of the accelerated ageing test at 1200 °C [18].

**Figure 10:**
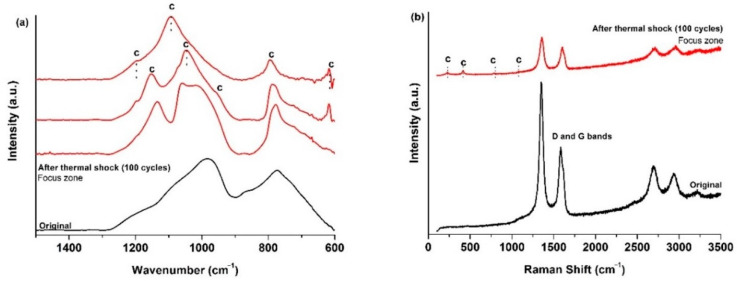
(**a**) ATR; (**b**) Raman spectra of the initial SiOC_d_ surface and, after 100 thermal shock cycles under concentrated solar radiation by Fresnel lens near the focus zone. c = cristobalite.

All these changes were also analysed by ATR and Raman spectroscopies. The ATR spectrum of the initial SiOC_d_ shows the bands related to Si–O and the SiOC mixed network, and a small band also appears at 880 cm^−1^ related to Si–C bonds [30]. After 100 thermal shock test cycles, in the focus zone, the spectrum changes and displays mainly the bands related to cristobalite with the presence of the most intense bands (1202, 1095, 1035, 619 cm^−1^), and other less intense bands (1156, 942 and 793 cm^−1^) [38] (Figure 10a). The Raman spectrum of the initial SiOC_d_ material shows D, G and second order bands [30] associated to the C_free_ phase with a noticeable reorganization with respect to SiOC_p_ which has a highly disordered C_free_ phase, basically due to the sintering temperature. The L_a_ value is 1.7 nm. After 100 cycles of the thermal ageing test, in the focus zone, C_free_ bands appear together with the cristobalite bands, where again, in addition to the presence of the most intense bands (231 and 415 cm^−1^) [38], the other less intense bands are also noticeable (782 and 1093 cm^−1^) [43] (Figure 10b).

Compared to the previous studies at 1200 °C [18], the modification of the surface of the SiOC_d_ material is much less obvious, as has been demonstrated by the ICM, SEM, ATR and Raman studies, and by the variation in both weight and density. Another point that supports this assumption is that the formation of a SiO_2_ layer is not found over all the material surface. In any case, the SiOC_d_ material supports the severe conditions of the thermal shock ageing test quite well (rapid heating at 32 °C s^−1^ and cooling at 27 °C s^−1^, from R.T. to 1000 °C, over 100 cycles), and the high temperature test at 1000 °C (100 cycles) [17], indicating the huge resistance at these experimental conditions and the suitability of SiOC_d_ material as a candidate for high-temperature solar receivers.

### 3.4. SiOC–SiC_d_

**Figure 11:**
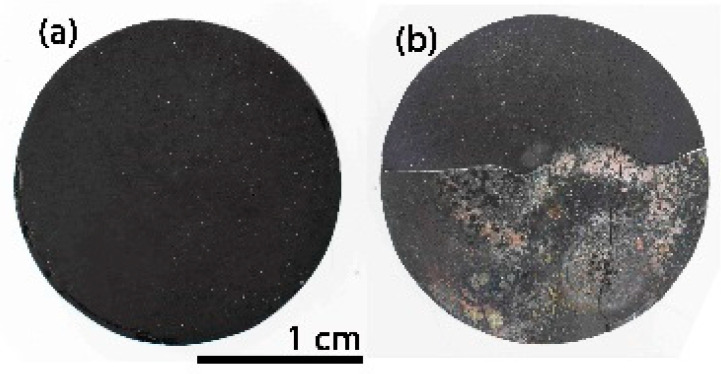
SiOC–SiC_d_ sample (**a**) before; (**b**) after 100 cycles of thermal shock testing under concentrated solar radiation by Fresnel lens.

The initial appearance of the dense black SiOC–SiC_d_ material is shown in Figure 11a. During the handling of the SiOC–SiC_d_ sample, it broke into two pieces before it was placed inside the stainless steel chamber; nevertheless, it was decided to perform the thermal ageing test on one of the pieces as the break did not occur while it was in service. The variation in temperature during the 100 cycles is shown in Figure S3d, and it can clearly be seen there is almost no variation during the thermal shock experiment.

**Figure 12:**
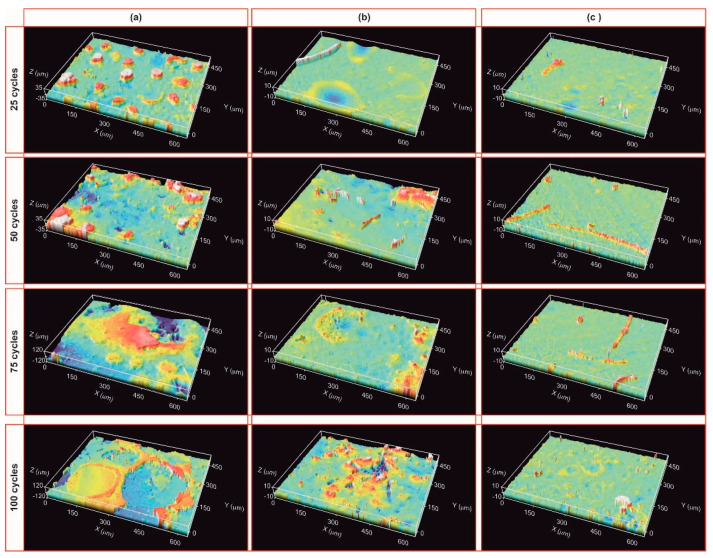
ICM images showing the surface of the SiOC–SiC_d_ sample after 25, 50, 75 and 100 cycles of the thermal shock test under concentrated solar radiation by Fresnel lens. (**a**) area nearest the solar radiation focus; (**b**) medium distance; (**c**) area furthest from the solar radiation focus.

After 25 cycles, the visual inspection does not reveal any change; however, the ICM study indicated major superficial changes, (Figure 12 and Table 1). The focus zone shows multiple peaks with a size of between 50 to 85 µm, wires greater than 100 µm and valleys over 130 µm (Figure 12a, 25 cycles). In the intermediate zone there are some valleys of up to 300 μm with a depth of <5.5 μm (Figure 12b, 25 cycles); and in the zone far from the focus, there are only a few peaks and valleys (Figure 12c, 25 cycles). It is important to note the presence of a cyan-green cover over the whole surface as was previously observed in the SiOC_d_ sample after 75 cycles (Figure 8b, 75 cycles), due to the formation of a SiO_2_ layer. R_a_ values increase particularly in the focus zone (Table 1). The transformation of the surface of the SiOC–SiC_d_ material must be related to the oxidation of C_free_ (3), SiOC (6); and also SiC according to reaction (7) [34,35].

SiC(s)+O_2_ (g) → SiO_2_ (s)+CO_x_(g) (7)

The massive degradation (i.e., two-piece fracture) of a SiC sample was observed earlier during the first cycle in a thermal ageing test performed on bulk SiC materials [18]. In this case, the fracture of the sample was explained in terms of the significant differences between the CTE of the SiC (6.6 × 10^–6^ °C) [10] compared to the protective SiO_2_ layer formed over the material (0.4 × 10^–6^ °C) [19] and the very severe conditions of the thermal shock test.

The focus and intermediate zones become more heterogeneous as the number of thermal shock cycles increases, showing the presence of more and larger peaks, valleys and wires (Figure 12a,b, 50–100 cycles). The focus zone has the highest roughness values after 75 thermal shock cycles (Table 1), and the size of the peaks and valleys is larger than in the case of 25 cycles Figure 12a, 75 cycles). This is probably due to the explosion of pristine grains and the melting and recrystallization processes. However, roughness values start to decrease from 75 to 100 cycles, probably indicating that the surface is smoothed by the formation of a denser and more homogeneous SiO_2_ layer all over the surface as was previously detected (Table 1) [18]. The size of peaks and valleys decreases to values of <250 μm (Figure 12, 100 cycles). The SiOC–SiC_d_ sample again broke while being positioned for the final cycles (from 75 to 100 cycles), but as this fracture did not occur in service, it was decided to carry out the last 25 cycles without taking it into consideration.

**Figure 13:**
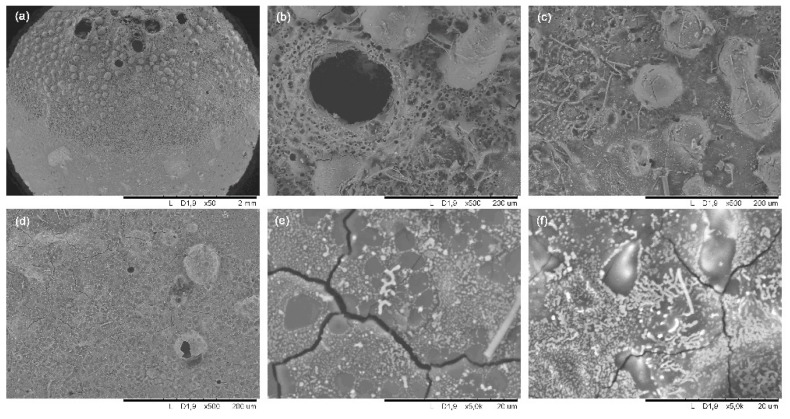
SEM images of the SiOC–SiC_d_ sample near the focus zone after 100 cycles of the thermal shock test showing: (**a**–**c**) large valleys, peaks and wires; (**d**) homogeneous layer; (**e**,**f**) cracks, fern-type and geometrical crystallites after 75 and 100 cycles, respectively.

SEM images of the SiOC–SiC_d_ sample after 75 and 100 cycles of thermal shock ageing are shown in Figure 13. The surface near the focus zone is deeply modified (Figure 13a) with the presence of large valleys, peaks and wires (Figure 13b,c) formed as consequence of the breakdown of C_free_, SiOC and SiC (reactions 3, 6 and 7 respectively). These images agree with those obtained by ICM (Figure 12). The whole surface of the SiOC–SiC_d_ material is homogeneously covered by a layer (Figure 13d), which, under higher magnification, reveals the presence of cracks and fern-type and geometrical crystallites, probably associated with different kinds of crystalline species of SiO_2_ and SiC, respectively. It is important to note that this layer becomes thicker and is homogeneously distributed all over the surface from 75 to 100 cycles (Figure 13e,f, respectively), supporting the assumption that the smoothing observed with ICM studies during these cycles could be directly related to the evolution of the SiO_2_ layer (i.e., denser and homogeneously distributed).

**Figure 14:**
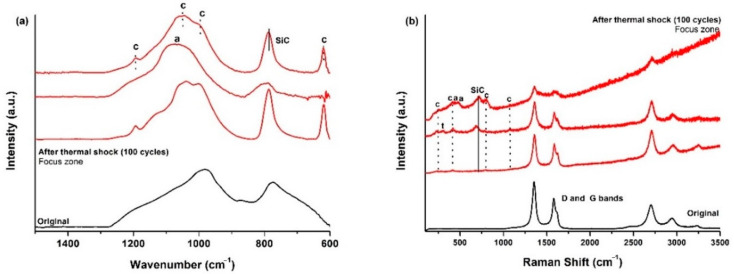
(**a**) ATR; (**b**) Raman spectra of the initial SiOC–SiC_d_ surface and, after 100 thermal shock cycles under concentrated solar radiation using a Fresnel lens near the focus zone. (t = tridymite, c = cristobalite, a = amorphous silica).

The ATR spectrum of the initial SiOC–SiC_d_ sample shows the bands related to Si–O, SiOC mixed network and Si–C bonds (880 cm^−1^) [30]. After 100 cycles of the thermal shock ageing test, in the focus zone, the spectra display the bands related to cristobalite (1202, 1095, 1035, 619 cm^−1^), amorphous silica (1200 and 1100 cm^−1^) [38], and a sharp peak at 780 cm^−1^ related to crystalline β-SiC [44] (Figure 14a). The Raman spectrum of the initial SiOC–SiC_d_ material shows D, G and second order bands [30] in the C_free_ phase with a similar degree of ordering to SiOC_d_. The L_a_ value is 2.7 nm. After 100 cycles of the thermal ageing test, in the focus zone, C_free_ bands appear with the cristobalite bands (229, 416, 700 and 1076 cm^−1^) [38,43], SiC (780 cm^−1^) [44] and small amounts of other SiO_2_ compounds are detected such as amorphous silica (441, 478 and 610 cm^−1^) [38] and tridymite (316 cm^−1^) [41] (Figure 14b). In summary, based on the experimental results, there is clear evidence of the formation of a denser and homogeneously distributed layer of SiO_2_ composed of cristobalite and amorphous silica, which coexists with the presence of crystallites of β-SiC and other silica species (i.e., tridymite).

In the case of dense materials, SiOC–SiC_d_ displays a greater modification of its surface when is compared with SiOC_d_ material although it was expected to experience better results due to the presence of SiC as reinforcement. This modification occurs faster in SiOC–SiC_d_ by the presence of larger L_a_ domains of C_free_ (L_a_ SiOC_d_ = 1.7 nm and L_a_ SiOC–SiC_d_ = 2.7 nm), which facilitate the evolution of gaseous species formed during the degradations of C_free_ (reaction 3) but also by the presence of domains of SiC which also produces both gaseous species and a layer of SiO_2_ (reaction 7).

After 100 cycles of accelerated ageing at 1000 °C, the visual inspection of the SiOC–SiC_d_ sample reveals the presence of a cover all over the surface (Figure 11b), which is caused by the formation of a protective silica layer [18,23]. The weight of the SiOC–SiC_d_ sample does not change, although its porosity varies from 4 to 0%. These data indicate that the evolution of gaseous species (i.e., O_2_ inwards and CO and CO_2_ outwards) must be very slow, as occurred in the SiOC_d_ sample, and the subsequent material densification could only be associated to the high temperature value used during the thermal shock ageing test. Accordingly, the very significant differences between the CTE of SiC (6.6 × 10^‒6^ °C) [10] with respect to the SiO_2_ layer formed over the material (0.4 × 10^‒6^ °C) [19] and the changes in the porosity of the material due to the very severe conditions of the thermal shock tests, contributes to the surface modification of the material. In any case, the SiOC–SiC_d_ material does not undergo massive degradation but rather a severe modification of its surface.

**Figure 15:**
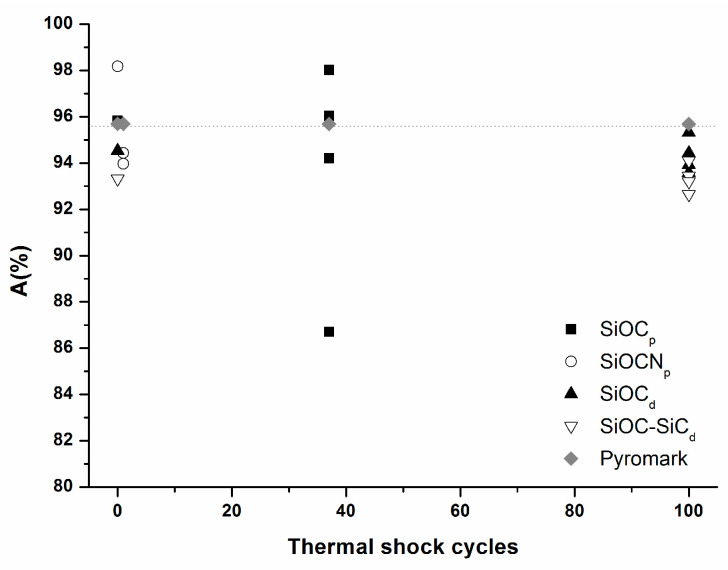
Variation of absorptance before and after thermal shock testing under concentrated solar radiation by Fresnel lens for SiOCN_p;_ SiOC_p;_ SiOC_d;_ SiOC–SiC_d_ compared with Pyromark^®^ paint.

The evolution of absorptance before and after the thermal shock tests was measured for all the samples, and was compared with Pyromark^®^ commercial paint. All the values are shown in Figure 15. It is well known that a material’s absorptance should be as high as possible for its application as a solar receiver. Considering only the initial sample A values, the highest value corresponds to the SiOCN_p_ sample (A = 98.2%) which also has a higher value than Pyromark^®^ paint (A = 95.7%). The other samples have slightly lower values, especially SiOC–SiC_d_ (A= 93.3%), but they closely match the values required for solar receivers (SiOC_p_, A = 95.8% and SiOC_d_, A = 94.5%). The samples were modified in different ways after the thermal shock tests, and the A values are very different depending on both the composition and porosity of samples. Porous samples were massively degraded, and they showed major variations in A values; so after one cycle for the SiOCN_p_ sample, the A value is 90.8, 94.0, 94.4 and 94.9%, and after 37 cycles for SiOC_p_ it is A = 86.7, 94.2, 96.0 and 98.0%. In the case of dense materials, and due to the formation of a protective silica layer which prevents the massive degradation of the material, the A values remain constant (SiOC–SiC_d_, A average (n = 4) of 93.3%) or decrease very slightly compared to the initial values (SiOC_d_, A average (n = 4) of 94.3%).

In view of all the results, the SiOC_d_ material is the most adequate to be used in the manufacture of solar receivers, due to: (a) its reasonable resistance to very harsh ageing tests at 1000 °C; (b) its surface durability, thanks to the formation of a protective silica layer (i.e., cristobalite and amorphous silica); and (c) its optical properties which remain quite constant (the absorptance value varied from 94.5 to 94.3%).

## 4. Conclusions

Four different ceramic materials have been studied under an accelerated thermal ageing experiment consisting of a thermal shock test using a Fresnel lens with very high heating (32 °C min^−1^) and cooling (27 °C min^−1^) rates, from 100 to 1000 °C, and a holding time of 10 min.

Porous materials (SiOCN_p_ and SiOC_p_) fail the thermal shock test and they were massively degraded during the first cycles. A protective layer, which should limit the diffusion of gaseous species inwards (O_2_) and outwards (CO_x_ or N_2_) was not formed and the fluxes can diffuse without restriction due to the highly porous microstructure and the very fast and severe conditions of the thermal shock test.

Dense materials (SiOC_d_ and SiOC–SiC_d_) withstand 100 cycles of thermal shock testing at 1000 °C. Their microstructure slows the diffusion of gaseous species both inwards (O_2_) and outwards (CO_x_) and forms a protective layer that prevents massive degradation. In SiOC_d_, the formation of a SiO_2_ layer is not obvious over the whole surface, indicating the huge resistance of the SiOC_d_ material against oxidation. The absorptance values only varied from 94.5 to 94.3%. Finally, in the SiOC–SiC_d_ material, a dense and homogenous layer composed of SiO_2_ and β-SiC crystallites was observed covering the whole surface and, the Absorptance values do not vary (93.3%).

In view of all these results, the high resistance to the thermal shock ageing tests at 1000 °C, and the best absorptance values of the SiOC_d_ material, point to this material as a very promising candidate for application in the manufacture of high-temperature solar receivers.

## Figures and Tables

**Table 1:** Profile roughness parameters of SiOCN_p_, SiOC_p_, SiOC_d_ and SiOC–SiC_d_ samples before and after the thermal shock test (R_0_ = initial roughness, R_1_ = roughness in the area nearest the solar radiation focus, R_2_ = medium distance and R_3_ = area furthest from the solar radiation focus). The roughness values correspond to R_a_: arithmetic average roughness value, expressed in μm.

Sample	R_0_	Number of Cycles	R_1_	R_2_	R_3_
SiOCN_p_	19.81	1–2	Failure		
SiOC_p_	5.26	25	6.73	6.40	6.12
		37–38	Failure		
SiOC_d_	0.20	25	0.24	0.22	0.18
		50	0.24	0.18	0.25
		75	18.9	0.34	0.28
		100	0.10	1.35	0.26
SiOC–SiC_d_	0.36	25	6.49	0.91	0.63
		50	8.13	1.50	0.70
		75	33.68	1.18	1.29
		100	27.01	2.22	0.86

## Data Availability

The data presented in this study are available on request from the corresponding author.

## Supplementary Materials

The following are available online at https://www.mdpi.com/1996-1944/14/4/1013/s1, Figure S1. SEM micrographs of the initial surface of (a) SiOCN_p_; (b) SiOC_p_; (c) SiOC_d_; (d) SiOC–SiC_d_. Figure S2. ICM images showing the initial surfaces of samples: (a) SiOCN_p_; (b) SiOC_p_; (c) SiOC_d_; (d) SiOC–SiC_d_. Figure S3. Temperature recordings during the thermal shock tests: (a) SiOCN_p_; (b) SiOC_p_; (c) SiOC_d_; (d) SiOC–SiC_d_. Figure S4. ICM images showing the surface of the SiOC_p_ sample after 25 cycles of the thermal shock tests: (a) nearest focus area; (b) middle zone; (c) furthest area from the solar radiation focus.
