# Supplementary material for: Silicon Oxycarbide and Silicon Oxycarbonitride Materials under Concentrated Solar Radiation
Source: Materials (Basel). 2021 Feb 21;14(4):1013. doi: 10.3390/ma14041013 (PMC7924838; doi:10.3390/ma14041013)
Supplement: Supplementary file 1 [file materials-14-01013-s001.pdf]

materials

MDPI

Supplementary Materials

Silicon Oxycarbide and Silicon Oxycarbonitride Materials under Concentrated Solar Radiation

M. Alejandra Mazo [1,*], Isabel Padilla [2], Aurora López-Delgado [2], Aitana Tamayo [1] and Juan Rubio [1]

[1] Ceramics and Glass Institute, CSIC, Kelsen 5, 28049 Madrid, Spain; aitanath@icv.csic.es (A.T.); jrubio@icv.csic.es (J.R.)
[2] National Centre for Metallurgical Research, CSIC, Av. Gregorio del Amo 8, 28040 Madrid, Spain; isapadilla@cenim.csic.es (I.P.); alopezdelgado@cenim.csic.es (A.L.-D.)
* Correspondence: sandra@icv.csic.es

Citation: Mazo, M.A.; Padilla, I.; López-Delgado, A.; Tamayo, A.; Rubio, J. Silicon Oxycarbide and Silicon Oxycarbonitride Materials under Concentrated Solar Radiation. *Materials* **2021**, *14*, 1013. https://doi.org/10.3390/ma14041013

Received: 15 December 2020
Accepted: 8 February 2021
Published: 21 February 2021

Publisher's Note: MDPI stays neutral with regard to jurisdictional claims in published maps and institutional affiliations.

Copyright: © 2021 by the authors. Licensee MDPI, Basel, Switzerland. This article is an open access article distributed under the terms and conditions of the Creative Commons Attribution (CC BY) license (http://creativecommons.org/licenses/by/4.0/).

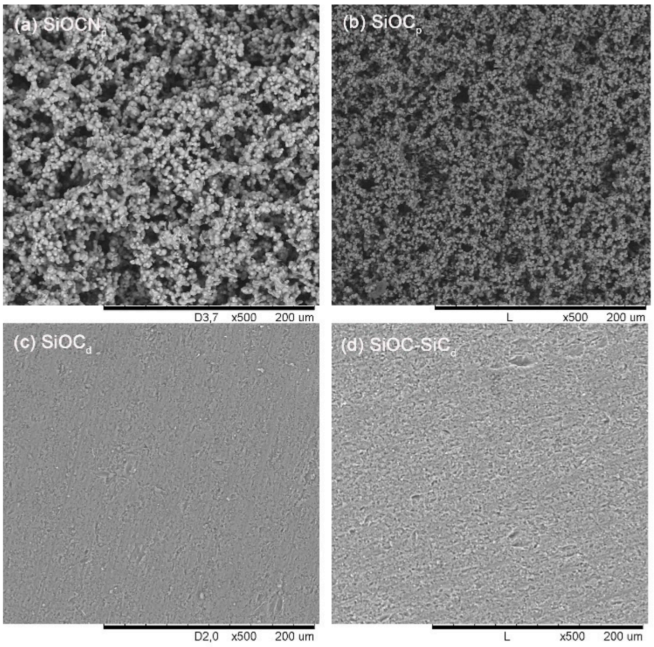

Figure S1. SEM micrographs of the initial surface of (**a**) $SiOCN_p$; (**b**) $SiOC_p$; (**c**) $SiOC_d$; (**d**) $SiOC–SiC_d$.

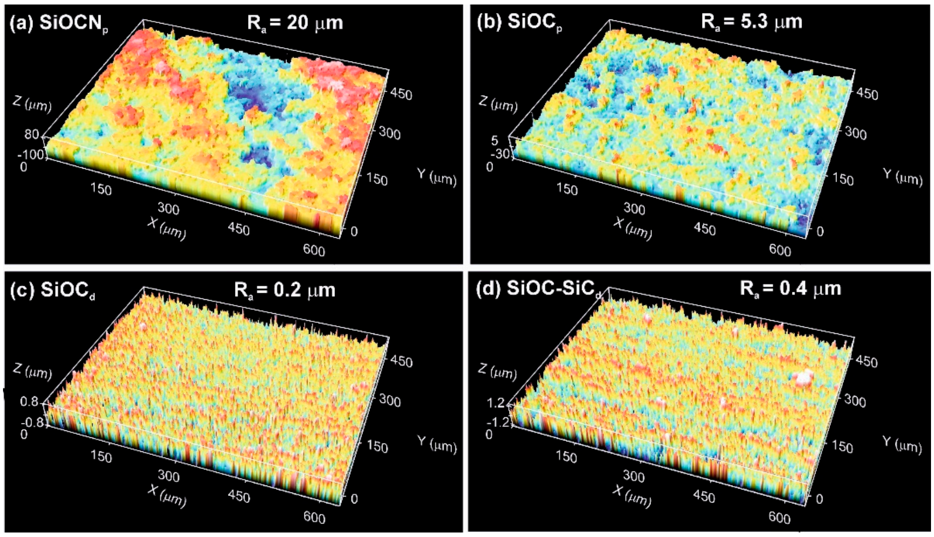

Figure S2. ICM images showing the initial surfaces of samples: (**a**) $SiOCN_p$; (**b**) $SiOC_p$; (**c**) $SiOC_d$; (**d**) $SiOC–SiC_d$.

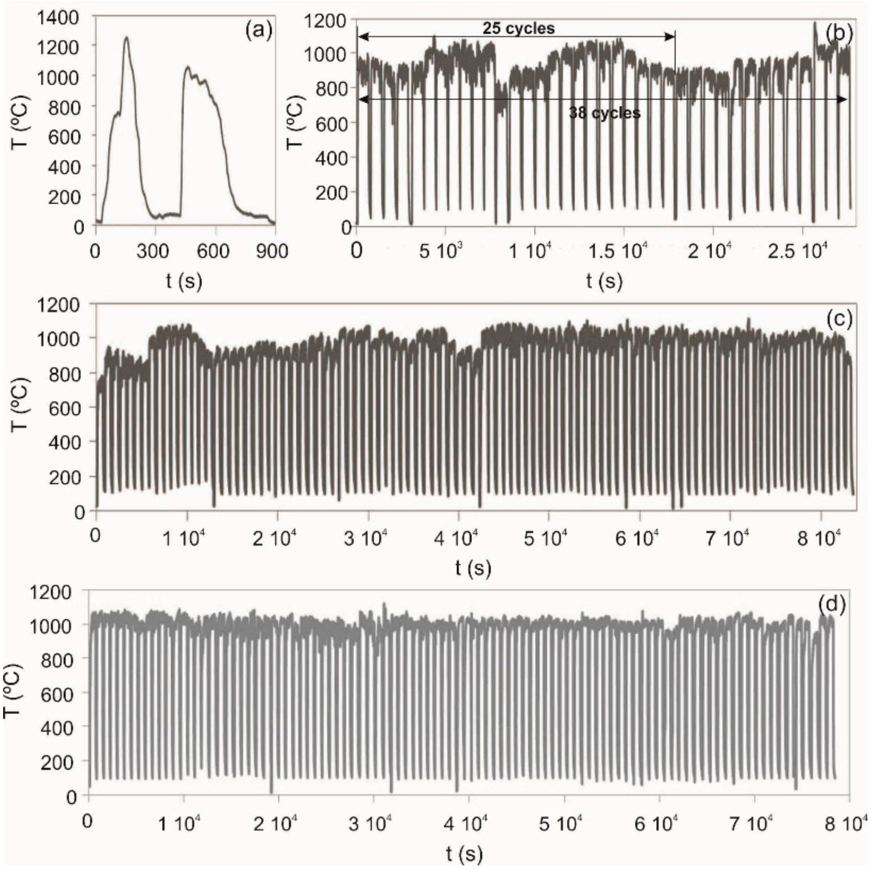

Figure S3. Temperature recordings during the thermal shock tests: (**a**) $SiOCN_P$; (**b**) $SiOC_P$; (**c**) $SiOC_d$; (**d**) $SiOC–SiC_d$.

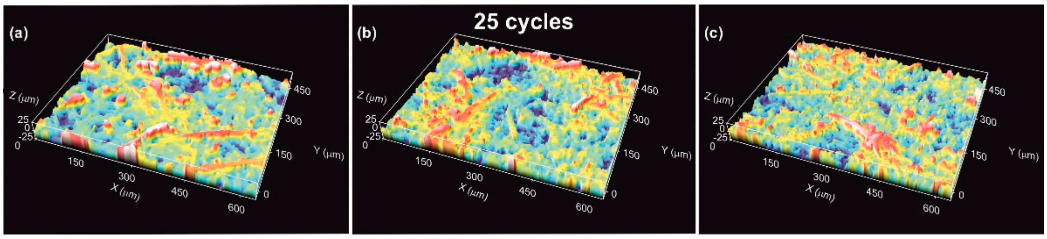

Figure S4. ICM images showing the surface of the $SiOC_P$ sample after 25 cycles of the thermal shock tests: (**a**) nearest focus area; (**b**) middle zone; (**c**) furthest area from the solar radiation focus.
